# Using Evidence and Coalitions to Scale-Up a National Early Education Initiative: The Case of Law 248/2015 in Romania

**DOI:** 10.3389/fpubh.2020.591421

**Published:** 2021-02-16

**Authors:** Maria Gheorghiu, Leslie Hawke, Joost de Laat, Cǎtǎlina-Alexandra Papari

**Affiliations:** ^1^Asociatia OvidiuRo, Bucharest, Romania; ^2^The AlexFund, New York, NY, United States; ^3^Utrecht Centre for Global Challenges, Utrecht School of Economics, Utrecht University, Utrecht, Netherlands; ^4^European Venture Philanthropy Association (EVPA), Brussels, Belgium

**Keywords:** scaling up, early childhood development, early childhood education, education system, education law, conditional cash transfers, roma children, evaluation

## Abstract

In 2015, Romania took an important step toward increasing disadvantaged children's participation in early education programmes through the passage of legislation creating a nationwide conditional cash transfer programme linked to preschool attendance. The programme was modeled on the incentive component of a 5-year pilot project “Every Child in Preschool” (“FCG”) initiated by Asociatia OvidiuRo (“OvR”), a small non-governmental organization. This paper explores how OvR used evidence from its pilot, global research, a quasi-experimental evaluation, collaboration with local authorities, and an intensive advocacy effort toward the legislative and executive branches of the national government to achieve the national scale-up of an early education initiative designed to create equal access in kindergarten among Roma and other impoverished, marginalized children.

## Introduction

Approximately 200 million children under the age of 5 living in low—and middle-income countries do not achieve their development potential as a result of poverty and deficient learning opportunities (1). Eurostat reports that 37.9% of the Romanian population is at risk of poverty and social exclusion, the highest share being recorded between 0 and 18 years old (2). These inequalities often begin before birth and grow during a child's early years (3).

In 2015, Romania took an important step toward improving access to early education for disadvantaged children through the passage, with the full support of all political parties^1^, of Law 248/2015. This legislation introduced a nationwide conditional cash transfer programme to encourage preschool and kindergarten participation of children from poor families (5). The law, intended to draw impoverished, predominantly Roma, children into existing early education programmes in rural Romania, was modeled after the “Every Child in Preschool” project, initiated in 2010 by Asociaţia OvidiuRo, a small non-governmental organization (NGO).

Using a combination of document analysis and unique interviews with stakeholders, this paper explores what led to the project's transformation into a national initiative.

## Literature Review

A large body of evidence exists on the importance of early childhood education (ECD) for later life outcomes [e.g., (6–8)], including recent special issues in (9), in the Annals of the New York Academy of Sciences (10), and the Archives of Disease in Childhood (2019) (11).

Each of the published articles underscores the importance of monitoring and evaluation: for example, to understand which children are not yet reached, which programmes work best for which sub-populations of children, where there might be bottlenecks, etc. However, monitoring and evaluation, and scientific evidence more broadly, does not automatically and universally lead to better designed and/or expanded ECD programs. In *Scaling early child development: What are the barriers and enablers?* (2019), part of the (11) series, ECD practitioners are reported to see monitoring and evaluation efforts as serving primarily scientific publication objectives, not project improvements or scaling.

Public policy interest and interest among civil society organizations in ECD has also been on the rise. The UN's Sustainable Development Goals (SDG) for 2030 includes SDG 4—Quality of Education, and focuses on lifelong learning opportunities for all, starting at birth and continuing through all phases of life (12). The importance of governance and the institutional frameworks around ECD are also increasingly recognized (13).

A case study by Ram (14) traces the steps that brought efforts, including ECD, to promote Roma inclusion across Eastern Europe prominently onto the agenda of the World Bank (15). Ram highlights that the World Bank's analytical work “was a prerequisite and driver of further action, though it alone was insufficient” (p. 578). The evidence used was complemented by other key factors such as: (i) individuals' efforts to elevate the issue; (ii) World Bank's external strong social networks providing expertise and funding; (iii) international context and the EU enlargement which imposed ex-conditionalities for Roma inclusion.

In the next sections, we explore the steps that led to the passage of Law 248/2015 and the 2020 amendments to the law.

## Methodology

We used a combination of document analysis and interviews with key stakeholders to identify the key factors that transformed a small NGO-run project into a national initiative, with particular attention to the role of monitoring, and evaluation in this process.

In spring 2019, we carried out ten face to face, telephone and written interviews with the main actors in the process of advocacy and legislative change. All the interviewees held a strategic or decisional position within the organization/institution they represent.

In addition to the co-founders of *Asociatia OvidiuRo* (OvR), Maria Gheorghiu, and Leslie Hawke, co-authors of this paper, we interviewed one current and one former OvR staff member who participated in developing the original pilot programme. We also interviewed senior representatives of other civil society organizations active in early education and acquainted with national policy dynamics: World Vision Romania, the Roma Education Fund, and Ready Nation Romania, an association of business leaders supporting early education. We interviewed a senior representative of the National Roma Agency (16), a public body, UNICEF Romania, the World Bank, and Up Romania, one of the companies that provided the food coupons that were used as conditional cash transfers. Lastly, we interviewed a former Secretary of State in the Ministry of Labor at the time Law 248/2015 was being considered in Parliament.

The interviews were complemented with the analyses of publications from a variety of civil society and public sources on the project, passage of Law 248/2015, and the international context.

## Legal and Institutional Developments on Early Childhood Education

More than four out of ten children in Romania (41.7%) are estimated to be at risk of poverty or social exclusion (2, 17, 18). The vast majority of Romanian children 4–5 years of age participate in early childhood education before the start of primary school [(19), cited in FRA (20)]. However, there are large disparities by ethnicity and socio-economic status. Participation is lowest among the large Roma minority, which by some estimates make up between 6 and 12% of the population, with approximately half living in rural areas (21)^2^. A survey of Roma communities across Eastern Europe found that 38% of Romanian Roma children aged 4–5 were participating in preschool compared to 80% of the general population (20)^3^.

Corresponding rates of early leavers from the education system among Roma are extremely high. The same 2016 FRA survey finds that 77% of Roma aged 18–24 years old do not continue their education beyond the lower secondary level. Among the general population the rate stands at 19%, still one of the highest in the EU (20).

**Figure 1:**
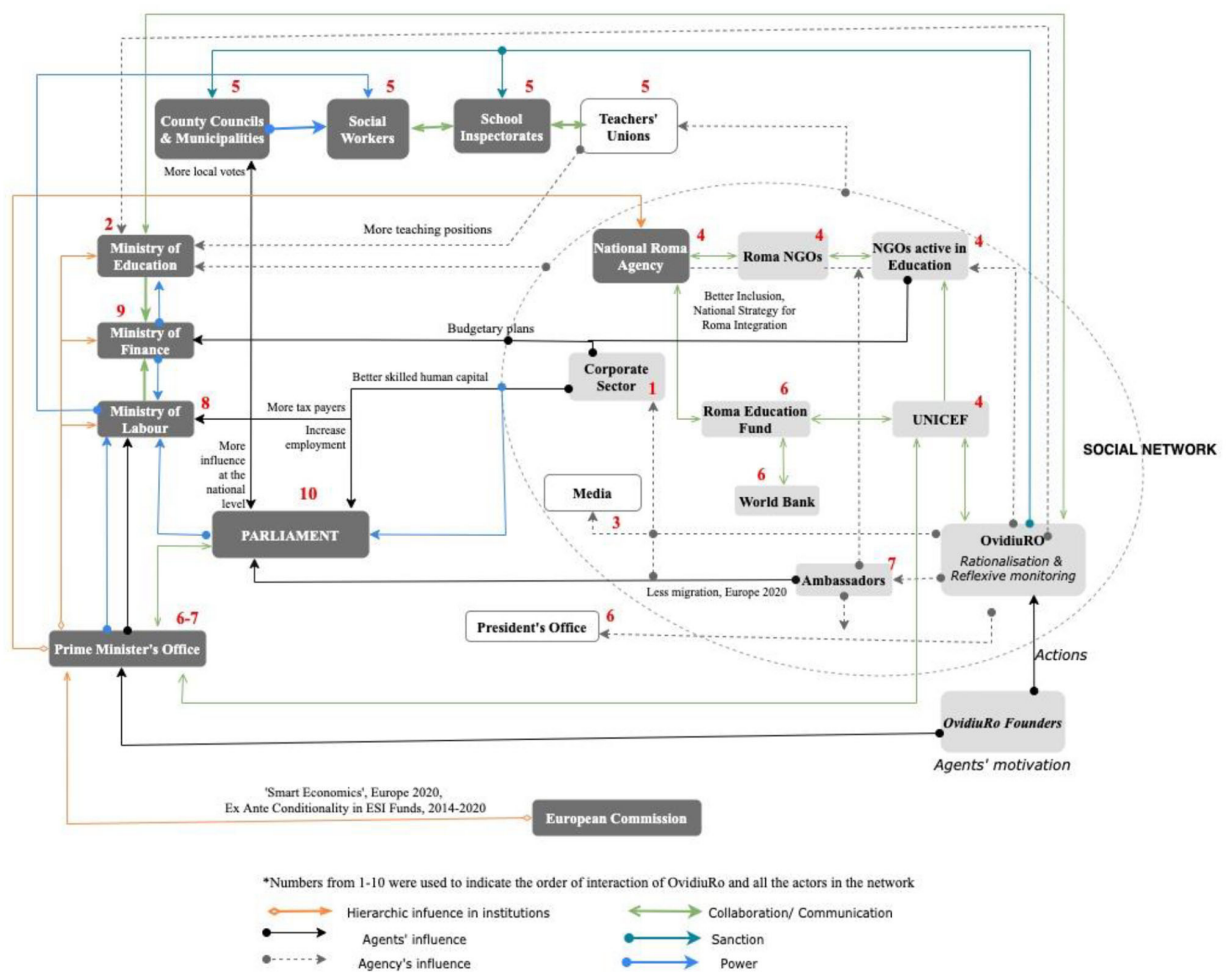
*Social Network Map—*Actors involved in the OvR project. Source: Compiled by the author based on data analysis of the interviews conducted and desk research.

Starting around 2010, calls for expanding ECD opportunities for vulnerable children as a way to address long term poverty and exclusion increased from public, civil society, and international organizations. Over time, this included a growing list of national organizations and agencies, including the Romanian Ministries of Education and Labor; international organizations, particularly, the European Commission, UNICEF, and the World Bank, and a range of national and international civil society organizations, such as OvidiuRo, Step by Step, World Vision, and FDSC (*Fundaţia pentru Dezvoltarea Societ*ăţ*ii Civile*). Some of these advocate more indirectly for early education as a means to promote equal opportunities for Roma children, such as the National Roma Agency, the Roma Education Fund, *Agenţia Împreun*ă, *Romani CRISS*, the Policy Center for Roma & Minorities; and even private sector networks like Ready Nation Figure 1.

The structure of early education, preschool and crèches, is provided by the National Law of Education (22). In 2011, the Romanian Government redesigned the national education system to focus on delivering a more inclusive and better-quality learning process, in part pushed by the EU Commission's request (23) to align national education policies with long-term EU jobs and growth strategy for 2010–2020 (24, 25).

The 2011 law extended the length of primary education by lowering the enrolment age from 7 to 6 (26). It also included a specific budget for investing in rehabilitation of schools in disadvantaged areas (27). However, the 2011 law did not address educational participation for children younger than 6, which remained both optional and costly for poor parents. Although pubic kindergartens do not charge tuition in Romania, there are fees for school materials and transport, and a noon meal for full-day programs^4^.

## Ovidiuro and the Evolution of “Every Child in Preschool”

### Context

Two of this paper's authors, Maria Gheorghiu, a Romanian primary school teacher, and Leslie Hawke, then a US Peace Corps volunteer, started an alternative education programme for unschooled children while working for the *Fundatia de Sprijin Comunitar* in Bacău, Romania in 2001, with an initial $60,000, 18-month grant from USAID. Starting in two dorm rooms in a public high school, the project soon grew by word of mouth to two nearby Roma enclaves, drawing in children, age 6–15, who were not registered for school, many of whom had aged out of the system. From the beginning, the programmes were always conducted in local schools, as Gheorghiu and Hawke's ultimate goal was to officially integrate the children into the public education system. They also wanted to make it clear to the authorities that the programme was truly educational and not merely a “day center.”

In 2004, Gheorghiu and Hawke founded Asociatia OvidiuRo, and expanded their project to a Bucharest elementary school (29, 30). Again, interest through word-of-mouth among school directors in poor communities led OvR to branch into kindergartens in neighboring counties, providing assistance in recruiting the community's unschooled children and training the teachers to work more effectively with children in need of remediation. OvR designed and implemented special classes, after-school programs, and summer sessions conducted in the schools by local teachers, but with training, and oversight from OvidiuRo.

**Figure 2:**
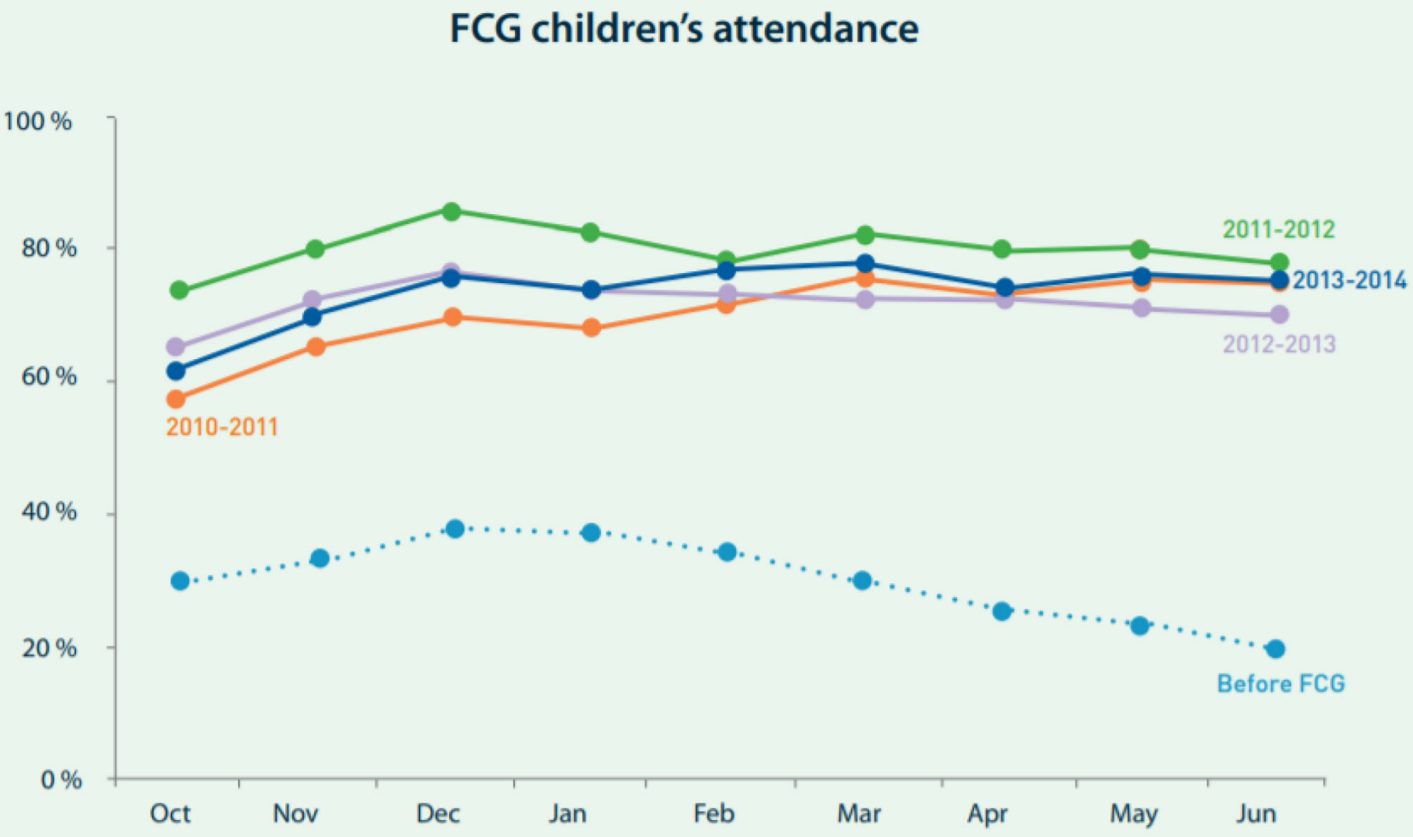
FCG children's attendance (graph extracted from the OvR's 4th Annual Report to Partners, Stakeholders & Investors, 2013–2014, p. 18) Source: Children's attendance to kindergarten before and after the FCG programme (32).

From the beginning, the founders had kept track, informally, through the teachers and social workers, of the children in the programs. By 2007, they were observing that the vast majority of the children did not stay in school more than 2 or 3 years (31). But on closer analysis, they observed that the younger a child had started in the programme, the greater the likelihood of their staying in school. This was true even for children in the same family Figure 2 (33).

They came to believe that none of the standard interventions could make a significant difference if a student's intellectual capacity had been stunted in early childhood [(34), p. 2]. Their first-hand experience in the field was corroborated by academic research published online (e.g., Steven Barnett^5^, James Heckman^6^, Harvard's Center for the Developing Child^7^), and World Bank reports^8^ regarding the importance of early intervention for disadvantaged children's long-term school success (39).

It was common knowledge that Roma children typically did not attend kindergarten (The parents, with little education themselves, tended to see kindergarten as superfluous and costly. And they were often subtly, or sometimes directly, discouraged from enrolling their children by the school administrators). Like OvR, the Ministry of Education and other NGOs were primarily focused on supporting at-risk elementary and secondary school students.

In 2010, OvR's founders decided to focus exclusively on “*gr*ă*diniţ*ă” (i.e., preschool and kindergarten) age children (40).

They had found, over the years, that it was not difficult to get impoverished parents, even traditional Roma, to register their children for school, but it was very difficult to get them to attend on a daily basis throughout the long frigid Romanian winter. A small 2007 UNDP grant had made it possible for OvR to test *tichete sociale* (“food coupons”) as an incentive to get impoverished parents to send their children to school everyday, and it had proven a quite effective attendance stimulant. Thus, in 2010, in partnership with the Ministry of Education, OvR began to use food coupons as a *preschool* attendance incentive.

A grant from The Alex Fund in NY^9^ allowed OvR to invite mayors across Romania to apply for assistance in getting every poor 3–5 years old child in their communities in *gr*ă*diniţ*ă by offering each family that qualified for social benefits, an additional monthly food coupon worth 50 lei (€10) if their child attended *gr*ă*diniţ*ă “every day.” (34).

The project, called *Fiecare Copil in Gr*ă*diniţ*ă (FCG), was a kind of joint venture between the local municipality and OvidiuRo. In order to qualify for financial aid from OvR, the Local Council was required to:

- Contribute €35 per year per impoverished preschool-aged child for shoes and clothes,
- Create a Local Action Group comprised of the mayor, an additional Local Council representative, a social worker, school director from each kindergarten; and
- Oversee door-to-door recruitment by the Local Action Teams (41).

OvR contributed food coupons worth €10/month per child, conditional on the child's daily attendance (and distributed by the teachers) to parents whose income was < €60/month. OvR also provided on-site consultation to the Local Action Group and training to the teachers and social workers.

To obtain the food coupons, monthly attendance reports had to be submitted by the school director to OvR. OvR's field staff were responsible for confirming the accuracy of the reports through frequent, and sometimes unannounced, community visits. A high rate of kindergarten participation resulted, with 85% of the 1,400 children in the programme qualifying for food coupons by their regular attendance in the 2011–2012 school year.

### Impact Evaluation

Gheorghiu and Hawke had been inspired by William Easterley's book, *The White Man's Burden*, which introduced them to the “conditional cash transfer” model, the work by the 2019 Nobel Laureates in Economics—Esther Duflo, Michael Kremer, and Abhijit Banerjee—and other affiliates of MIT's Jameel Poverty Action Lab (JPAL), and the importance of independent impact evaluations. Easterly's observations about the ineffectiveness of most international aid efforts rang very true to their own observations. The success of programmes in Mexico (Opportunidades) and Brazil (Bolsa Familia) combined with their own small-scale experience with food coupons in 2007, accelerated their interest in getting their project evaluated independently to bolster support of their long-range national expansion goals. Toward that end, Maria attended a JPAL Seminar in London the summer of 2009.

By 2012 anecdotal reporting by mayors, school directors, and teachers indicated that children who had participated in FCG were better prepared for primary school than similar children who had not attended preschool. But OvR needed more objective evidence of this in order to:

- Convince other communities to adopt the programme,
- Persuade county governments to allocate funds to expand it throughout their county, and
- Support future advocacy efforts to get the Government to expand the programme nationally.

The OvR team also wanted to demonstrate the program's efficacy to UNICEF Romania, which was also actively promoting early education to the Romanian Government (27, 42, 43), and had previously funded OvR programmes. However, while UNICEF Romania supported the end-goal—to increase preschool participation among poor and disadvantaged children (33, 44)—UNICEF objected to the instrument—conditional cash transfers. OvR argued that its direct experience in poor rural communities had shown that the prevalence of disincentives to sending poor children to early education (e.g., lack of transportation, clothes, shoes, and school supplies, coupled with often unwelcoming attitudes of the school authorities) made it necessary to provide positive associations in order to get the parents' attention and ultimately, their buy-in (45). The food coupon incentives clearly increased disadvantaged children's regular preschool attendance. And that, OvR maintained, was the necessary first step to increasing their successful integration in primary school. Keen to objectively demonstrate the model's real-world impact to any potential detractors, OvR's leaders sought independent verification.

In 2012 a team of academic researchers, supported by evaluation funding from the World Bank's Strategic Impact Evaluation Fund, agreed to partner with OvidiuRo on a prospective randomized control trial (RCT) (46, 47). In the end, OvR lacked the financial resources to expand the project to the required number of newly identified and randomly assigned communities, preventing implementation of the RCT. Ultimately, a different team of academic researchers conducted a quasi-experimental evaluation that compared communities with the project with non-treated communities sharing similar socio-economic characteristics.

“The results indicated that the FCG programme has a significant impact on both enrollment and attendance in preschool and lower primary school. With 50 lei per child per month, it offers an effective way to incentivize poor parents to send their children regularly to preschool. This behavior change seems to translate to an increased willingness to attend school well-beyond the end of the program.We therefore strongly recommend to scale up the programme to the national level.” [(48), p. 6].

This scholarly, scientific approbation turned out to be extremely helpful in convincing local authorities in new regions to adopt the intervention as well as convincing potential corporate sponsors to invest. It was also valuable, when meeting with members of Parliament, to be able to reference the study and include its summary of results in the package of supporting materials.

### Preparing for National Scale-Up

From the very beginning in the early 2000's, OvR's executive director, Maria Gheorghiu, who had spent 15 years teaching in Bucharest schools before partnering with Hawke, met frequently with key Ministry of Education officials to keep them apprised of OvR's activities, making sure their efforts were in sync with the Ministry's priorities, building support for scaling up.

In 2008, OvidiuRo's founders set the year 2020 as their goal for expanding their programme “*Fiecare Copil in SCOALA*” to the national level (Hawke, annual meeting personal notes, 2008). “*Fiecare Copil in Scoala*” became “*Fiecare Copil in GRADINIŢA*” in 2010 when it became emphatically clear that the earlier children started, the better their educational outcomes, with late school starters almost never catching up or staying in school more than 2 years.

A 2010 partnership agreement with the Ministry of Education had given OvR formal permission to carry out FCG activities in public schools. It also allowed the use of the Ministry's logo on project materials, which was important to gaining the attention of county inspectorate administrators, and facilitated access to kindergartens for monitoring purposes (32).

In 2013, as a result of Gheorghiu's repeated urging, the Ministry of Education formed an inter-ministerial Early Education Working Group with the Ministry of Labor and OvR to lay a foundation for transforming FCG into a national program. One of the tasks of this group was to clarify the legal framework so that the conditional food coupons could be allocated by public authorities.

FCG was most successful in communities where the school director, social workers and municipal personnel participated in the monitoring process (49). On the whole, given the lack of resources and the voluntary nature of the project by local institutions with competing priorities, consistent involvement and oversight by OvR was common. But since the long-term goal was for the programmes to operate independently, in 2014, OvR introduced a process to wean programmes from management-dependence on OvR by giving well-functioning programmes more autonomy in local decision-making.

By fall 2015, FCG was reaching 2,500 children in 45 communities in 12 counties (50) largely funded by corporations operating in Romania. Corporate leaders were sensitive to the issue of workforce shortages and responsive to OvR's argument that investing early would have a high long-term return through better skilled human capital (Ready Nation, personal communication, April 17, 2019) a message that fit with their CSR objectives. Corporate donors also required far less paperwork than government or international organization grants—and a phone call to the CSR director was usually all that was required if they needed to rearrange line items in the budget.

Significantly, the project predecessor to FCG, *Fiecare Copil in Scoala*, was rejected for an EU structural funds grant in 2008 because there was no precedent for a conditional incentive project (Leslie Hawke, personal communication, July 2020). The only government-generated grant that FCG received was a “European Economic Area” (EEA) grant in 2014^10^ which provided the funds for FCG to extend to six new communities, with the explicit goal of getting the county councils to cover the cost of food coupons after the grant period. This goal turned out to be crucial to the scale-up because it forced OvR's leaders to address the legal obstacles to transitioning to public funding. It effectively forced them to petition Parliament in 2015 to approve the concept of food coupons linked to daily attendance as a legitimate government-approved expense, in order to pave the way for the counties to finance the food coupon incentives.

### Advocacy Process in Scaling-Up the Conditional Cash Transfer Component

Efforts to promote FCG as a national programme caught traction when in 2011 the American Ambassador to Romania, Mark Gitenstein, advised OvR to convene a meeting of influential ambassadors to Romania on the subject of early education. Nine European ambassadors, the UN representative to Romania, and Ambassador Gitenstein participated in the first annual meeting of the *Ambassadors' Early Education Initiative*” in 2012. Subsequently, American, British, German, Israeli and Norwegian ambassadors visited FCG rural project sites, experiencing first-hand Romania's hidden Roma poverty (Hawke correspondence, 2020). Although the group was entirely informal, having the attention and support of European officials who often spoke with Romanian government authorities, helped publicize both the importance of early education in general, and the FCG project in particular, among high level Romanian officials. It also facilitated opportunities for OvR's leaders to talk about the programme at embassy events.

Over the spring of 2015, Gheorghiu and Hawke met with political party leaders and Senate committee chairs. Right before the summer recess, legislation designed to ease the way for counties to finance FCG unanimously^11^ passed the Senate. Gheorghiu and Hawke began to meet with various Chamber of Deputies members over the summer and hired an aide to set up appointments and follow up with County Council presidents and members of parliament.

One of the most important advocates for expanding FCG was the Secretary of State from the Ministry of Labor at that time, Codrin Scutaru, who had visited OvR's programmes early on and was personally committed to helping expand FCG. He took a leading role in drafting the original bill and persuading an important Senator to sponsor it, and arranged for his boss, the Minister of Labor, to visit an FCG project in her home county in the spring of 2015. In the summer of 2015, Scutaru resigned his post at the Ministry of Labor to run the McGuire-Woods public affairs office in Bucharest. Subsequently, the McGuire-Woods team also advocated for the legislation among politicians from the different parties.

### Passage of Law 248/2015

To the astonishment of the OvR team, instead of allowing the county governments to utilize county public funds for FCG, the Romanian Parliament voted in October 2015 to make the parent incentive component a national early education incentive programme, allocating €11 million in the 2016 state budget for this purpose. Having gained commanding support from all the political parties in Romania, the final Chamber of Deputies vote was 289 votes in favor, one against, and five abstentions (51).

Law 248/2015 introduced a food coupon incentive of 50 lei (~€10) per month conditional on the child's daily attendance. Families with children ages 3–5 were eligible, provided family income was not higher than 284 lei (€58.66) per family member per month.

Two weeks later, economic and social problems erupted in Romania and led to a major political upheaval that resulted in the resignation of the Prime Minister and each Government Minister. Before resigning, the Minister of Labor succeeded in allocating money from the national budget as one of her last official duties (Reality Check, personal communication, April 18, 2019).

Still, before the law could take effect, “secondary legislation,” the process of turning the law into a specific set of rules and regulations, had to be drafted and agreed-upon by both the Ministry of Labor and the Ministry of Education, since both agencies' personnel would be involved in the execution. At Gheorghiu's prodding, the secondary legislation was finalized in January and the law went into effect in February 2016 (Hawke, personal communication, July 2020).

Concerned that the government had not set aside resources to inform the counties or local authorities about the legislation, OvR's entire staff of 12 took to the road in three teams to meet with authorities in all 41 counties in February and March of 2016, informing and instructing local implementation teams in the proper procedures for implementation and taking note of concerns that were broached at the town hall style meetings. Separate meetings were held with the County Council and Prefects (50).

### Implementation Challenges

The OvidiuRo team, well-aware of the amount of oversight and communication required to establish a high-functioning programme was of two minds when the legislation passed^12^. On one hand, this was an extraordinary opportunity to exponentially increase the number of disadvantaged children in kindergarten. On the other hand, it was difficult for an NGO with 12 full-time staff members and a budget of €750,000 to drive the expansion of an early education incentive programme in 45 rural communities into one active in thousands of municipalities across Romania.

As part of its effort to facilitate implementation of the law, the OvR team visited 500 rural kindergartens in 38 counties in 2016 and 2017. Obstacles to the seamless roll-out of the programme, like delays in coupon distribution and conflicting interpretation of the regulations, were relatively easy to ameliorate. More serious obstacles ranged from a shortage of classroom space and social workers to indifferent mayors and school directors. To contribute to solving the classes (school infrastructure) limitations, OvidiuRo offered small grants (approximated at 2,500 euros) to communities that requested help in transforming other spaces, such as storerooms or smaller rooms not used by schools, into kindergarten classes (52). At the same time, the number of children in classes was supplemented, with additional 2–3 children per each level of study. To overcome the social workers' shortage, OvR team initiated many campaigns of promotion with their small number of staff resources, as well as involving teachers and school directors in the process of informing families about the programme^13^.

Tracking the number of children benefitting from the legislation proved to be difficult. In the first year (2015–2016), the Ministry of Finance estimated that 79,000 children benefited. In 2017, coupon suppliers tallied 42,000 children and the Ministry of Education 33,700 (OvR, personal communication, June 4, 2019 and Reality Check, personal communication, April 18, 2019). OvR considered the data collection from the three food coupon suppliers to be the most accurate, estimation (UpRomania, personal communication, April 15, 2019) as the government's data was inconsistent: the data provided by the Ministry of Labor, Ministry of Education, and Ministry of Finance were different one from each other and they often resulted in considerable disparity. The coupon suppliers' data came from their actual invoices to communities, being 100% reliable and providing a good overview (53–55).

### The Role of Reality Check

In 2018, former OvR field director, Alina Seghedi, started “Reality Check” a separate NGO with the mission of improving public policies through applied monitoring (56). One of its early activities was an evaluation (49) of Law 248/2015's implementation in order to identify ways to assist localities where programme enrollment was low. They found that impediments to success included parents' difficulty in applying for the programme, misunderstandings about the methodology, the small incentive amount compared to the costs of getting children to school every day, and the lack of social workers or teachers proactively recruiting children. Based on these findings, Reality Check led a successful effort to amend the law. In April 2020, Parliament passed four amendments to take effect January 2021. The main changes are that children in families receiving social welfare payments (SFA) will automatically be eligible, the food coupon incentive doubles to 100 lei per month (€21), and mayors and school directors are required to organize information and enrolment campaigns twice annually^14^^,^^15^.

## Conclusions

In 2015, Romania took an important step toward increasing disadvantaged children's participation in early education programmes through the passage of legislation creating a nationwide conditional cash transfer programme linked to preschool attendance.

In the years leading up to the law's passing, public authorities at both the local and national levels knew that early school abandonment was rampant in many poor rural communities, but did not have a long-term strategy nor had dedicated the resources to tackle it. Many communities had benefitted sporadically from short-term outside grants to address this, but most of these projects terminated as soon as the outside funding ran out, and therefore, never lasted long enough to take root in the community or to make any measurable difference in student outcomes^16^.

In hindsight, the absence of a scientific randomized control trail measuring the project's impact did not turn out to be a major hurdle to the passage of a national law. Still, project monitoring and quasi-experimental impact evaluation evidence, and (international) scientific evidence more broadly, was important in several ways. At the local level, data from the project documenting take-up of the food vouchers among beneficiaries and increased participation in kindergartens were essential in the early phase to mobilize interest and participation beyond the initial set of communities. This local evidence also helped support the narrative to get buy-in at the national level, alongside a body of international scientific evidence on early learning and conditional cash transfers.

More generally, OvR sought to address the challenge of low kindergarten participation among Roma and other disadvantaged children in a systemic approach that emphasized:

- **Significant investment from local authorities:** The local council was required to contribute at least €35 per child for school supplies as a precondition to participate in the programme;
- **Long-term commitment from OvR:** It was made apparent, from its continual communication, frequent on-site visits, and reliable food-coupon provision, that OvR intended to help develop, and sustain FCG;
- **Medium-term management transition plan:** In 2014, OvR introduced a process to wean programmes from management-dependence on OvR by giving well-functioning programmes more autonomy in local decision-making;
- **Measuring results:** Although a randomized control trial was not financially feasible, OvR conducted extensive project monitoring and supported an independent impact evaluation to support its advocacy. However, the independent research about the effectiveness of the programme was not crucial in scaling-up the FCG by passing legislation in 2015, but more to amend the law in 2019, by using the evidence on implementation challenges and possible solutions as a key argument in convincing MPs and government officials.
- **Positive word of mouth:** Over a 5-year period, informal communication among educators, social workers, mayors, and school inspectors facilitated the organic growth of the programme and created a positive word of mouth that eventually reached the ears of legislators; and
- **Strategic advocacy of the programme, and of conditional cash transfers more generally, to the executive and legislative branches:** An intensive advocacy effort, first by OvidiuRo, and then augmented independently by others, dovetailed with support from ambassadors and other public figures.

The Law 248/2015 was a major step forward in the effort to prepare children who had been neglected by the system to succeed in school. To ensure effective implementation of the law, OvR's leadership worked closely with the Ministries of Labor and Education on the “secondary legislation,” and OvR organized a country level outreach to inform and support communities in implementing the law.

In the opinion of OvR's founders, the biggest gain from the legislation was that it sent the unambiguous message to public authorities, teachers, and parents that early education was important. The local authorities were now expected to facilitate the registration and attendance of the community's most marginalized children. This was an entirely new mandate. The programme also sent the message to poor parents that their children were welcome in *grǎdiniţ.ǎ*. According to OvR's founders, the value of the food coupons was largely symbolic.

## Data Availability Statement

The raw data supporting the conclusions of this article will be made available by the authors, without undue reservation.

## Ethics Statement

Ethical review and approval was not required for the study on human participants in accordance with the local legislation and institutional requirements. Written informed consent for participation was not required for this study in accordance with the national legislation and the institutional requirements. Written informed consent was obtained from the individual(s) for the publication of any potentially identifiable images or data included in this article.

## Author Contributions

C-AP initiating research, gathering data, writing the first versions of the paper, editing and finalising the paper, and coordinating the writing process. JL editing paper and writing the literature review, contributing with insights on data, and reviewing the initial work of the corresponding author. LH contributing with insights on the scale-up of the pilot project/processing data, editing the paper, and contributing to the writing process. MG reviewing data and contributing with insights on the scale-up of the pilot project. All authors contributed to the article and approved the submitted version.

## Conflict of Interest

The authors declare that the research was conducted in the absence of any commercial or financial relationships that could be construed as a potential conflict of interest.

## Footnotes

^1^The final vote in the Chamber of Deputies was 289 votes in favor, one against, and five abstentions. (4)

^2^A National Report Regarding Social Protection and Social Inclusion estimated that almost 25% of people under the age of 18 are of Roma ethnicity. (Bucharest, September 2006).

^3^Comparable to figures from Slovakia (34%) and the Czech Republic (34%), although lower than Bulgaria (66%) and Hungary (91%); https://fra.europa.eu/sites/default/files/fra_uploads/fra-2016-eu-minorities-survey-roma-selected-findings_en.pdf.

^4^2018 amendments to article 16 of the 2011 Law stipulate the full extension of mandatory general education – including preschool - by 2023 (28).

^5^Barnett, (35).

^6^Heckman, (36).

^7^Harvard Center for the Developing Child.

^8^E.g., (37, 38).

^9^2010-2011 OvR Annual Report, p. 13.

^10^from Norway, Iceland and Liechtenstein, and administered by FDSC.

^11^117 for, 0 opposed, with 2 abstentions. Investor Report: From Pilot to Policy: Changing Romania's Educational Future−3,000 Kindergartens at a Time, October 2016.

^12^When Gheorghiu, in the final Labor Committee hearing, questioned the wisdom of expanding from 45 programmes to 4,500, the committee chair said, “The poorest counties, which need it the most, can't afford it. We have to go national.” (Hawke, personal communication, July 2020).

^13^The amendments to the law, which passed in April 2020, require mayors and school directors to organize program information and enrollment campaigns twice a year.

^14^https://www.alexfund.org/fcglawevolution/

^15^Out of 329 MPs, 327 were present, 326 voted in favor and 1 against.

^16^OvR 5th Year Report to Partners, Stakeholders & Investors, 2015.
